# Dendritic Cells Utilize the Evolutionarily Conserved WASH and Retromer Complexes to Promote MHCII Recycling and Helper T Cell Priming

**DOI:** 10.1371/journal.pone.0098606

**Published:** 2014-06-02

**Authors:** Daniel B. Graham, Douglas G. Osborne, Joshua T. Piotrowski, Timothy S. Gomez, Grzegorz B. Gmyrek, Holly M. Akilesh, Adish Dani, Daniel D. Billadeau, Wojciech Swat

**Affiliations:** 1 Department of Pathology and Immunology, Division of Immunobiology, Washington University School of Medicine, St. Louis, Missouri, United States of America,; 2 Department of Immunology, Division of Oncology Research and Schulze Center for Novel Therapeutics, Mayo Clinic College of Medicine, Rochester, Minnesota, United States of America; Istituto Superiore di Sanità, Italy

## Abstract

Immature dendritic cells (DCs) maintain a highly dynamic pool of recycling MHCII that promotes sampling of environmental antigens for presentation to T helper cells. However, the molecular basis of MHCII recycling and the cellular machinery that orchestrates MHCII trafficking are incompletely understood. Using a mouse model we show that WASH, an actin regulatory protein that facilitates retromer function, is essential for MHCII recycling and efficient priming of T helper cells. We further demonstrate that WASH deficiency results in impaired MHCII surface levels, recycling, and an accumulation of polyubiquitinated MHCII complexes, which are subsequently slated for premature lysosomal degradation. Consequently, conditional deletion of the *Wash* gene in DCs impairs priming of both conventional and autoimmune T helper cells *in vivo* and attenuates disease progression in a model of experimental autoimmune encephalitis (EAE). Thus, we identify a novel mechanism in which DCs employ the evolutionarily conserved WASH and retromer complex for MHCII recycling in order to regulate T helper cell priming.

## Introduction

The unique ability of DCs to sample their environment and present exogenous antigens on surface MHCII is essential for priming antigen-specific naive T lymphocytes [1]–[3]. At steady state, immature DCs survey their environment by constitutive macropinocytosis and phagocytosis [4]–[6] while maintaining a dynamic intracellular pool of MHCII for antigenic peptide loading [7]–[10]. Although immature DCs are highly efficient at antigen acquisition and processing, peptide-MHCII (pMHCII) complexes are labile as a result of rapid recycling and degradation [8], [9], [11]–[13]. Upon stimulation by pathogen-associated molecular patterns or inflammatory mediators, DCs undergo a rapid maturation that results in the down regulation of antigen uptake and pMHCII recycling [8], [11], [12], [14], [15]. As a result, DC maturation leads to the accumulation of surface pMHCII complexes bearing antigenic peptides encountered at the time of pathogen exposure or inflammatory insult [8], [16]. Consequently, DCs are adept at persistent display of antigenic pMHCII complexes on the plasma membrane to promote stable interactions with antigen-specific T cells.

Several previous studies have shown that efficient recycling of pMHCII in DCs is critical for preventing premature degradation of pMHCII complexes and that recycling facilitates the display of antigenic peptides to T helper cells [8], [15], [17]–[23]. Using multidimensional RNAi screening, Paul *et al*. found multiple proteins that play a role in MHCII gene expression and MHCII transport to the surface [24]–[28]. Studies examining MHCII transport from the plasma membrane through the endosomal network have mainly concentrated on the internalization of MHCII. Internalization of MHCII from the plasma membrane is thought to involve clathrin-mediated endocytosis, but it may also occur in a clathrin-independent manner involving lipid rafts and the activity of Arf6 GTPases [8], [29]–[31]. Once MHCII enters the endosomal compartment, its trafficking itinerary is not well characterized. From early endosomes, MHCII is thought to be transported to a peptide loading compartment referred to as the MHC class II-enriched compartment, which is likely a late endosome or multivesicular body [15], [20], [22]. Here, a critical sorting step occurs in which MHCII is either recycled or targeted for degradation via ubiquitination of a conserved lysine residue in the MHCIIβ chain [8], [18], [19]. In DCs, March1 ubiquitinates MHCII [17], [20]–[23], thus ostensibly marking it for recognition by the ESCRT complex, which transports cargo into luminal vesicles within the multivesicular body [7], [9], [15], [28], [32]–[35]. From here, MHCII is degraded in either lysosomes or multilamellar bodies [19], [24], [27], [28]. Efficient recycling of MHCII spares it from the degradation pathway. We previously reported that the immunoreceptor tyrosine-based activation motif (ITAM) signaling pathway is required for efficient MHCII recycling and antigen presentation [23], [24]. However, the cellular machinery responsible for recycling has not been defined.

MHCII is known to enter the endosomal compartment, but how it recycles and escapes delivery to the lysosome remains unclear [27], [30]. The retromer complex was shown to recycle mannose-6-phosphate receptors and G-protein coupled receptors from the endosomal compartment, thus sparing them from lysosomal degradation [18]–[20], [24], [36], [37]. In this context, the retromer complex is traditionally known for its role in directing retrograde trafficking of endosomal membrane components to the trans Golgi network [23], [38], [39], however emerging evidence indicates that retromer components also direct endosomal cargo to the plasma membrane [24], [26]–[28], [32], [38]. In both cases, the retromer functions in cargo recognition and endosomal tubulation with the vacuolar protein sorting subunits (VPS26, VPS29, and VPS35) functioning as a coat complex, while the sorting nexin subunits (SNX1, SNX2, SNX5, and SNX6) drive endosomal tubulation [7], [9], [28], [32], [33], [38], [40].

The discovery of the WASP family protein WASH was a major advancement in understanding actin-dependent receptor trafficking from the endosome. WASH functionally coordinates retromer activity with actin cytoskeletal remodeling to maintain endosomal structure and facilitate receptor trafficking by the retromer [7], [9], [10], [28], [33], [40]. WASH itself contains a VCA domain, which recruits and activates the Arp2/3 complex, leading to polymerization of branched actin networks on endosomes [7], [10], [41]. Although WASH is constitutively active with respect to Arp2/3-mediated actin polymerization *in vitro*, it is maintained in an inactive state in intact cells by the WASH regulatory complex (SHRC), which is comprised of Strumpellin, SWIP, FAM21, and CCDC53 [7], [10], [40]–[42]. In addition to maintaining WASH in an inactive state, the WASH complex member FAM21 recruits the complex to the retromer through a direct interaction with VPS35 [40], [42], [43]. WASH activity toward Arp2/3 is subsequently stimulated by K63-linked ubiquitination by the retromer-recruited TRIM27-MAGEL2-Ube20 ubiquitin ligase complex [9], [11], [43]. Here, we have identified a novel mechanism in which DCs employ WASH and the evolutionarily conserved retromer machinery for MHCII recycling from the endosomal compartment back to the plasma membrane. Loss of WASH or the essential retromer component VPS35 impairs MHCII recycling and antigen presentation by DCs.

## Materials and Methods

### Mice

Generation of mice with conditional *Wash* allele has been described previously [9], [11], [44]. In brief, the endogenous *Wash* gene was floxed (*WASH^fl/fl^*) by standard gene targeting technology and bred to LysM-Cre [44], [45] (Jackson Labs), Vav-Cre (Gift from Dr. Thomas Graf, Albert Einstein College) or CD11c-Cre mice [45], [46] (Jackson Labs). OT-II mice were a gift from H. Virgin (Washington University, St Louis, MO). Mice were bred on a C57BL/6 background.

### Ethics

All animal procedures were performed in accordance with institutional guidelines and approved by the Animal Studies Committee at Washington University School of Medicine in St Louis, MO (20120250) and Mayo Clinic Institutional Animal Care and Use Committee in Rochester, MN (A25413).

### DC cultures and shRNA knockdown

Bone marrow derived DCs (BMDCs) were cultured in GM-CSF as previously described [23], [46]. Short hairpin RNA constructs cloned into pLKO.1 lentiviral vector (the RNAi Consortium) were obtained from the Genome Institute at Washington University. Constructs targeting luciferase (AGTACTTCGAAATGTCCGTTC) or murine VPS35 (GCTGTCACCAAAGAGTTACTA) were transfected into HEK 293T cells along with pCMV-dR8.91 and VSV-G constructs to generate lentiviral particles. At 24 hours post- transfection, cell culture media was exchanged, and lentiviral supernatants were harvested 24 hours later. In parallel, BMDCs were harvested after two days in culture and “spinfected” with fresh lentiviral supernatant for 60 min. BMDCs were resuspended, diluted 1∶4 in fresh media, and cultured for 2 more days. At this time point, cultures were selected in puromycin (4 µg/ml) for the last 2 days of culture.

### MHCII surface retention

To determine MHCII cell surface retention in DCs, we turned to an assay we previously developed [7], [23], [42]. The MHCII cell surface retention assay was set up such that identical replicate samples were used for each time point after chase. Thus, we were able to monitor change in MHCII surface expression relative to time 0 (or untreated). Indeed, during extensive assay development, we have determined this approach to be the most quantitative and robust protocol relative to other iterations we tested. Furthermore, data from the surface retention assay are consistent with biochemical and cell biological approaches we have pursued [7], [23], [42]. In brief, BMDCs were treated with or without Brefeldin-A (GolgiPlug protein transport inhibitor BD Biosciences at 1∶1000). At time 0 and after 5 hours in Brefeldin-A, cells were stained with anti-CD11c-APC (BD Biosciences) and anti-I-A^b^-FITC (BD Biosciences) and analyzed by flow cytometry. The mean fluorescence intensity of MHCII at 5 hours was determined and expressed as a percent of the mean fluorescence intensity at time 0.

### Immunofluorescence (IF)

BMDCs were cultured on #1.5 LabTek II eight-chambered coverslips (Nunc) for at least 30 minutes to allow firm adhesion. Cells were fixed by addition of ice-cold fixative (4% paraformaldehyde and 0.5% glutaraldehyde in PBS) and incubated for 30 min at room temperature in the dark, followed by permeabilization with 0.2% Triton X-100 in PBS for 15 min. Cells were then cultured with 10 µg/ml primary antibodies (Abs) in IF buffer (TBS plus human serum cocktail) overnight at 4°C in a humidifier chamber. Primary Abs consist of a mouse monoclonal anti-I-A^b^ (Biolegend), rat polyclonal anti-LAMP1 (BD Biosciences), and rabbit polyclonal anti-WASH and anti-VPS35 antibodies [7], [16], [42] diluted in IF buffer. Following 5–6 washes in PBS, cells were incubated with secondary Abs (1∶500 dilution in imaging buffer) for 1 hr at room temperature. After 5–6 washes with PBS and addition of Hoechst 33342 nuclear stain, SlowFade Gold antifade reagent (Molecular Probes) was added to the wells. Images were obtained with an LSM-710 laser scanning confocal microscope with a 100X/1.4 Oil Plan-Aprochromat objective lens using ZEN software (Carl Zeiss). Each image represents an individual slice taken from a z-stack comprised of several slices at 0.25 µm depth.

### MHCII endocytosis assay

To specifically track the localization of surface-derived MHCII after endocytosis, we performed an IF directed endocytosis assay. BMDCs were cultured on #1.5 LabTek II eight-chambered coverslips as described above. After 30 min culture, the cell media was replaced with serum-free DMEM containing 10 µg/ml anti-I-A^b^ (Biolegend) and then incubated at 37°C for 30 min to allow internalization. Cells were then prepared for immunofluorescence using the protocol described above.

### Image Analysis

Co-localization was assessed by Pearson's correlation coefficient and overlap coefficient using ZEN software (Carl Zeiss). Fluorescent puncta and size were measured using single slice images and *Analyze Particles* in Image J (National Institutes of Health: http://rsb.info.nih.gov/ij/).

### MHCII half-life

BMDCs were surface biotinylated (5×10^6^ cells/100 µl in PBS pH 8.0) with Sulfo-NHS-SS-Biotin (Pierce Biotechnology) at a concentration of 0.5 mg/ml for 30 min on ice. After washing in PBS 10 mM glycine, cells were chased at 37°C for the indicated time points in complete media. Cells were then lysed for 5 min on ice in 1 ml of lysis buffer (1% NP-40, 150 mM NaCl, 50 mM Tris-HCl, 2 mM EDTA, supplemented with complete protease inhibitors (Roche), pH 7.2). Protein A/G sepharose (Pierce Biotechnology) was added to lysates along with 2.5 µg of anti-MHCII (clone M5/114) for immunoprecipitation at 4°C overnight. Samples were washed 3 times in lysis buffer, eluted in non-reducing 2× Laemmli sample buffer, and resolved by PAGE on a 10% gel. Proteins were then transferred from the gel to PVDF membranes (Millipore) that were then blotted with streptavidin-HRP (Southern Biotechnology) and developed using ECL substrate (GE Healthcare). After developing the blots, Image J software was used for densitometric measurement of band intensity (in units of mean integrated pixel density).

### MHCII recycling assay

BMDCs of the indicated genotypes were surfaced labeled with an anti-I-A^b^-APC antibody (BD Biosciences) for 30 min, washed in DC culture media (RPMI-10% FBS + GM-CSF + IL-4), and incubated for 30 min to allow internalization. Cells were then spun down and resuspended in FACS buffer stripping solution (PBS containing 2% BSA Fraction V [Sigma Aldrich] and 0.1% NaN_3_, pH 1.5) for 10 min on ice and washed in stripping solution. Cells were then washed in cold FACS buffer (pH 7.4 PBS containing 2% BSA Fraction V [Sigma Aldrich] and 0.1% NaN_3_) and resuspended in DC culture media. Resuspended DCs were then incubated for 0, 10, 20 and 40 min to allow resurfacing of the internalized I-A^b^. Following incubation, cells again were spun down and resuspended in FACS buffer stripping solution for 10 min on ice and washed in stripping solution. Cells were then washed, resuspended in 500 µl FACS buffer and analyzed by flow cytometry using a FACSCanto II flow cytometer (BD Biosciences). Data were analyzed using FlowJo 8.8.7 software (Tree Star, Ashland, OR). The percentage of recycled I-A^b^ was measured using the equation (T_0_ – T_x_)/T_0_×100. T_0_ represents the mean fluorescence of cells following the second acid strip at time zero and T_x_ is the mean fluorescence intensity of cells stripped at each time point. The acid stripping method was adapted from Sullivan *et al*. [16], [23].

### I-A^b^ surface expression

BMDCs *WASH^fl/fl^* Vav-Cre^+/+^ and control Vav-Cre^+/+^ mice were scraped from cultures and resuspended at 10^6^ cells/ml in FACS buffer. Cells were stained for 30 min at 4°C with anti-I-A^b^-APC. After a wash in FACS buffer, cells were resuspended in 500 µl FACS buffer and analyzed using a flow cytometry. Data were analyzed using FlowJo 8.8.7 software.

### MHCII Ubiquitination

Dendritic cells (15×10^6^/sample) were lysed for 10 min on ice in 1 ml of lysis buffer (1% NP-40, 150 mM NaCl, 50 mM Tris-HCl, 2 mM EDTA, 20 mM N-ethylmaleimide, supplemented with complete protease inhibitors (Roche), pH 7.2). Protein A/G sepharose (Pierce Biotechnology) and 3 µg of anti-MHCII (clone M5/114) were added to lysates for immunoprecipitation at 4°C overnight. Samples were washed 3 times in lysis buffer, eluted in Laemmli sample buffer by boiling, resolved by PAGE on a 10% gel, and transferred to PVDF membranes (Millipore). Membranes were then sequentially blotted with antibodies specific for ubiquitin (clone P4D1, Santa Cruz Biotechnology) or MHCII βchain (clone KL295, ATCC). Primary antibodies were detected with HRP-coupled anti-mouse IgG antibodies (Zymed) and developed using ECL (GE Healthcare).

### Antigen Presentation Assays

CD4^+^ OT-II T cells were purified from spleens and lymph nodes by negative selection with MACS beads (Miltenyi Biotec) and then labeled with CFSE (Vybrant CFDA SE cell tracer kit, Invitrogen) according to manufacturer's instructions. T cells (100×10^3^) were mixed with DCs (20×10^3^) in round-bottom 96-well plates with the indicated doses of OT-II peptide (Ova323-339) (a gift from P.M. Allen, Washington University). Alternatively, BMDCs were pulsed with OT-II peptide (100 nM) in serum free media for 1.5 hours at 37°C, followed by thorough washing, and cultured at the indicated cell number with OT-II T cells (100×10^3^) for three days. Subsequently, T cells were stained with anti-CD4-APC (clone GK1.5, Becton Dickinson) and analyzed on a FACSCaliber flow cytometer (Becton Dickinson) with FlowJo software. Proliferation was determined by cell count of CD4-positive CFSE-positive cells.

### Ovalbumin Immunizations

*WASH^fl/fl^ CD11c-Cre* mice and controls (6–8 weeks old) were immunized with 10 nmoles of the OVA323-339 peptide emulsified with complete Freund's adjuvant (Sigma Aldrich). Mice were injected subcutaneously into the hind footpad, and at seven days after immunization, the popliteal draining lymph nodes were harvested and processed into single cell suspensions for subsequent analyses.

### ELISPOT Assays

MultiScreen – IP sterile 96-well filtration plates with 0.45 µm hydrophobic high protein binding Immobilon-P membrane (Millipore) were pre-wet with 70% ethanol, washed with PBS, and then coated with anti-mouse IL-2 antibody (Becton Dickinson) at a final concentration of 5 µg/ml in PBS. Subsequently, lymph node cells from immunized mice were cultured on MultiScreen plates with MOG35-55 or OVA323-339 peptide (in triplicate) at a concentration of 2×10^5^ cells/well. After overnight culture, plates were extensively washed with water and blocked with 1% BSA in PBS for 1 hr at room temperature. Subsequently, biotinylated anti-mouse IL-2 capture antibody (2 µg/ml) and streptavidin-AKP (Becton Dickinson) were applied for 1 hr at room temperature. Plates were then washed with water prior to addition of Sigma Fast BCIP/NBT (Sigma Aldrich) detection reagent. After drying the plates overnight, the number of spots was calculated with an ELISPOT reader provided by Cellular Technology Ltd. using dedicated Immunospot Software.

### Experimental Autoimmune Encephalitis

Mice were immunized with 50 µg of MOG peptide (myelin oligodendrocyte glycoprotein amino acids 35–55) emulsified in IFA containing 50 µg *Mycobacterium tuberculosis* (strain H37RA) by subcutaneous injection. At the time of immunization and three days later, mice received 300 ng of pertussis toxin (List Biological Laboratories) intravenously. Subsequently, mice were evaluated daily and scored for development of EAE. Clinical scores of 1–5 were assigned as follows: grade 1 =  Tail weakness, grade 2 =  hind limb weakness sufficient to impair righting, grade 3 =  one limb plegic, grade 4 =  hind limb paralysis, grade 5 =  moribund.

### Statistical Methods

Data are expressed throughout as mean + standard deviation. Data sets were compared using the two-tailed unpaired Student's t-test. Statistical analysis (Student's t-test and column statistics) and graphing were performed using Prism 4 (GraphPad Software). Differences were considered statistically significant when p<0.05.

## Results

### Retromer maintains MHCII plasma membrane levels in DCs

**Figure 1:**
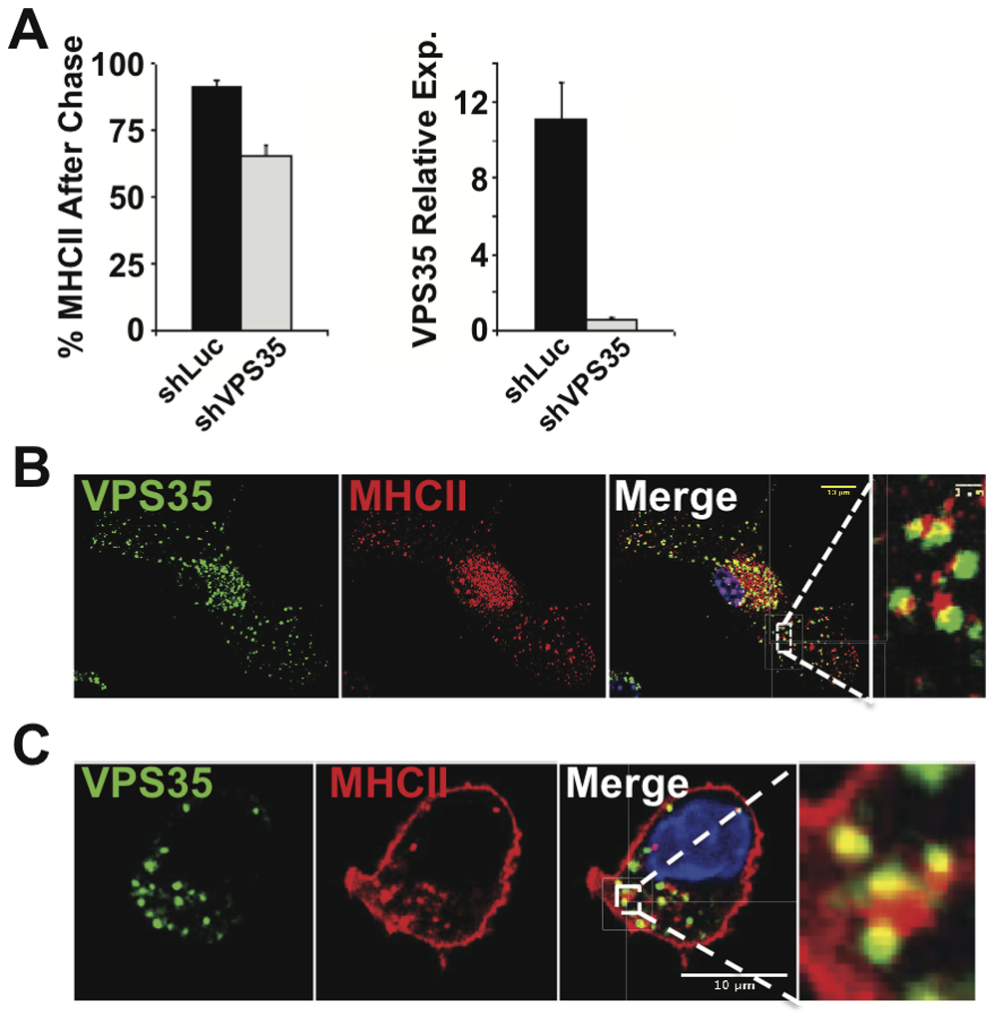
VPS35 localizes with MHCII and Vps35 is required in MHCII cell surface retention. (A) BMDCs were transduced with shRNA constructs targeting VPS35 (shVPS35) or luciferase (shLuc) as a control. Cells were treated with the Golgi transport inhibitor Brefeldin-A for a 5-hour chase, and MHCII cell surface expression was determined by flow cytometry (see Materials and Methods section for details). The percent of initial MHCII remaining on the plasma membrane after chase was calculated for three independent samples and plotted as mean ±s.d. To confirm efficient knockdown, VPS35 expression relative to β-actin was determined by quantitative PCR. (B) BMDCs were fixed and labeled with antibodies against MHCII and VPS35 for microscopic analysis. (C) Following the MHCII endocytosis assay, BMDCs were fixed and labeled with anti-VPS35. Zoomed images are demarcated by the white box and dashed lines in the adjacent image. For each condition, >20 individual cells were imaged. Images were collected with 100× oil objective. Scale bars, 10 µm and 1 µm.

We have previously developed an *in vitro* approach to systematically identify novel molecules involved in recycling of MHCII complexes on the surface of immature DCs [23], [24], [27], [33]. In this approach, surface expression of newly synthesized MHCII is blocked by inhibitors of Golgi transport (Brefeldin-A), permitting direct analysis of MHCII recycling rates on the plasma membrane by flow cytometry. After treatment with inhibitors, surface expression of MHCII is monitored over time to ascertain the rate of its disappearance, which is referred to as MHCII surface retention. To discover novel factors required for MHCII recycling, we used a lentiviral shRNA knockdown screen of candidate molecules involved in endosomal trafficking. We first focused on the retromer complex, which is traditionally known for its role in directing retrograde trafficking of receptors to the Golgi apparatus. In light of emerging evidence indicating that the retromer may also direct endosomal cargo back to the plasma membrane [24], [27], [28], [33], we examined if retromer function is required for MHCII recycling by depleting it in DCs using shRNA. Efficient knockdown of VPS35 was confirmed by qPCR prior to measurement of MHCII retention on the plasma membrane (Figure 1A, right panel). Strikingly, VPS35 knockdown in DCs led to a dramatic (40%) reduction in MHCII surface retention over the course of 5 hours with Brefeldin-A treatment (Figure 1A, left panel). In contrast, DCs transduced with control shRNA constructs or mock-transduced DCs exhibited a modest reduction (20%) of MHCII surface retention (Figure 1 and data not shown). Furthermore, we observed the accumulation of MHCII in a VPS35-enriched endosomal compartment (Figure 1B) consistent with the retromer being involved in the trafficking of MHCII. To confirm that the localization of MHCII with VPS35 is specific to endosomal recycling, IF was performed using an endocytosis assay. Prior to permeabilization, cells were incubated with anti-MHCII antibody to stain the plasma membrane localized MHCII, and then incubated to allow internalization. This procedure allowed us to specifically track cell surface MHCII and the endocytic pathway involved in it's recycling. Significantly, we observed similar localization of MHCII with VPS35 using the endocytosis assay (Figure 1C) suggesting that the retromer complex is likely involved in the recycling of plasma membrane MHCII through a trafficking route that appears to bypass the Golgi apparatus.

### WASH deletion results in an intracellular accumulation of MHCII

**Figure 2:**
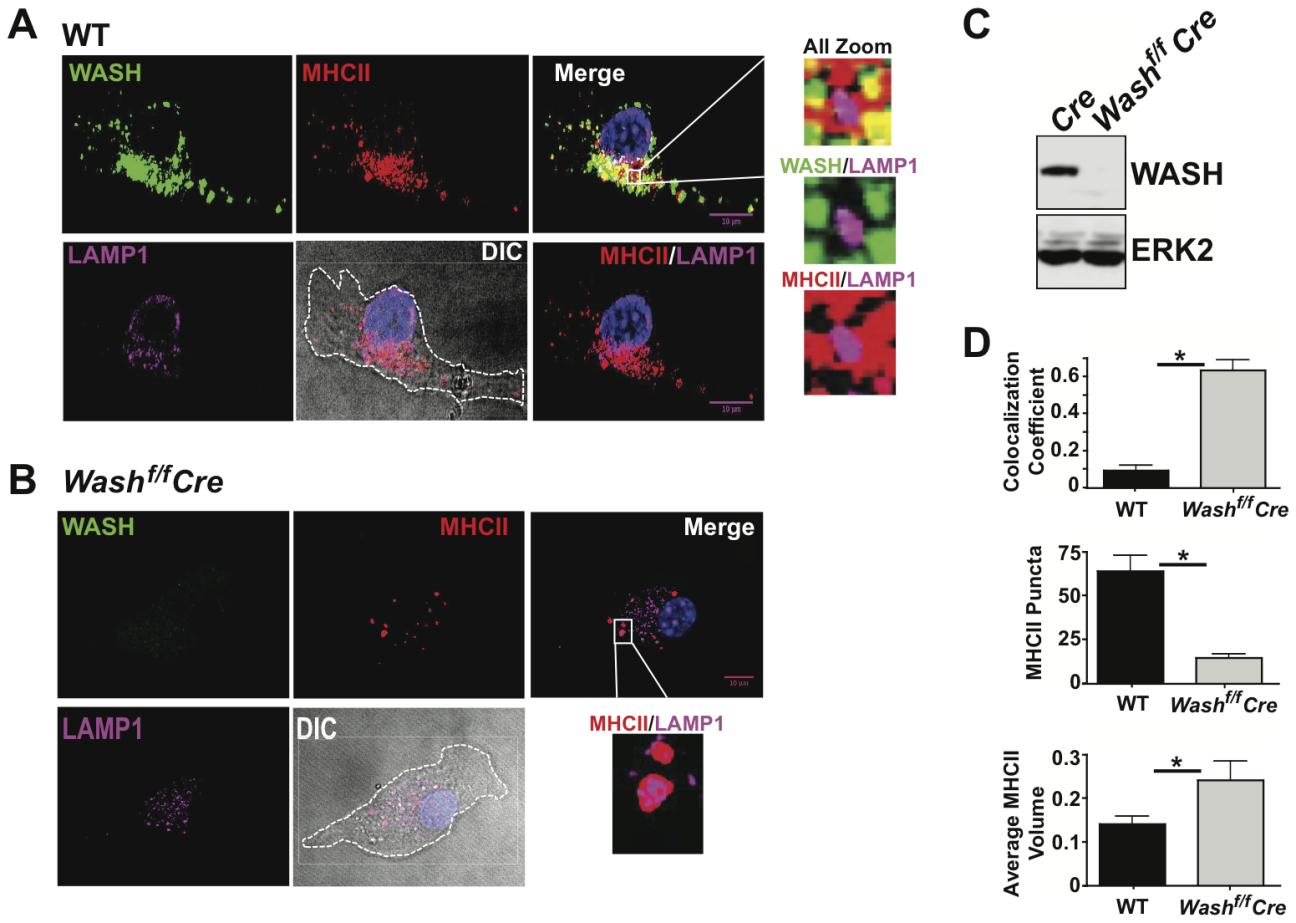
WASH localizes with MHCII in DCs. BMDCs from (A) Cre and (B) *WASH^f/f^* Cre were fixed and labeled with anti-MHCII antibody and WASH for microscopic analysis. (C) Protein lysates prepared from Cre and *WASH^f/f^* Cre BMDCs were immunoblotted for WASH and ERK2 (as a loading control). (D) Images from A and B were analyzed for MHCII co-localization with LAMP1 using Pearson's co-localization coefficient. MHCII puncta number and size were quantified by Image J. Zoomed images are demarcated by the white box and lines toward the adjacent image. Differential interference contrast (DIC) images were used to demarcate the outline of the cell. For each condition, >20 individual cells were imaged. Images were collected with 100× oil objective. Scale bars, 10 µm.

Recent evidence indicates that the WASH complex is involved in regulating the trafficking of several proteins that are sorted by the retromer [28]. Notably, while we clearly detect MHCII staining on both the plasma membrane and endosomes, VPS35 localized directly adjacent to MHCII on intracellular vesicular structures (Figure 1B and 1C). Consistent with previously published studies in various cell lines [7]–[10], we found that DCs similarly showed a unique distribution pattern of WASH characterized by punctate spots apposed to the early endosomal marker EEA1 (Figure 2A and Figure S1A and S1B). Strikingly, co-staining revealed that internalized MHCII complexes colocalized with WASH and EEA1 in DC endosomes (Figure 2A, zoom and Figure S1A and S1B).

To determine whether MHCII recycling in DCs required WASH, we used a conditional gene targeting approach in which Cre recombinase-mediated deletion of *lox*P-flanked *Wash* alleles (*Wash^fl/fl^*) terminates expression [8], [9], [11]–[13]. Mice harboring the floxed *Wash* gene were crossed with CD11c-Cre or Vav-Cre transgenic mice, in which the expression of Cre is restricted to DC- or hematopoietic-lineage cells, respectively. As an alternative strategy we generated LysM-Cre^+/+^ *Wash^fl/fl^* mice in which expression of Cre and subsequent deletion of the *lox*P-flanked *Wash* gene occurs in myeloid cells, including macrophages and monocyte-derived DCs. Importantly, in the absence of WASH, all DC subsets developed normally *in vivo*, and bone marrow derived DC cultures (BMDCs) expanded in GM-CSF with similar kinetics as wild type cells (Figure S2 and data not shown). Significantly, WASH-deficient DCs showed a loss of WASH protein by both IF and immunoblot (Figure 2B and 2C). Interestingly, in the absence of WASH, MHCII staining was substantially diminished at the plasma membrane and fewer overall intracellular MHCII positive puncta were observed (Figure 2B and 2D). Thus, we conclude that MHCII co-localizes within endosomes where WASH and VPS35 are accumulated.

### WASH prevents the localization of MHCII to lysosomes

**Figure 3:**
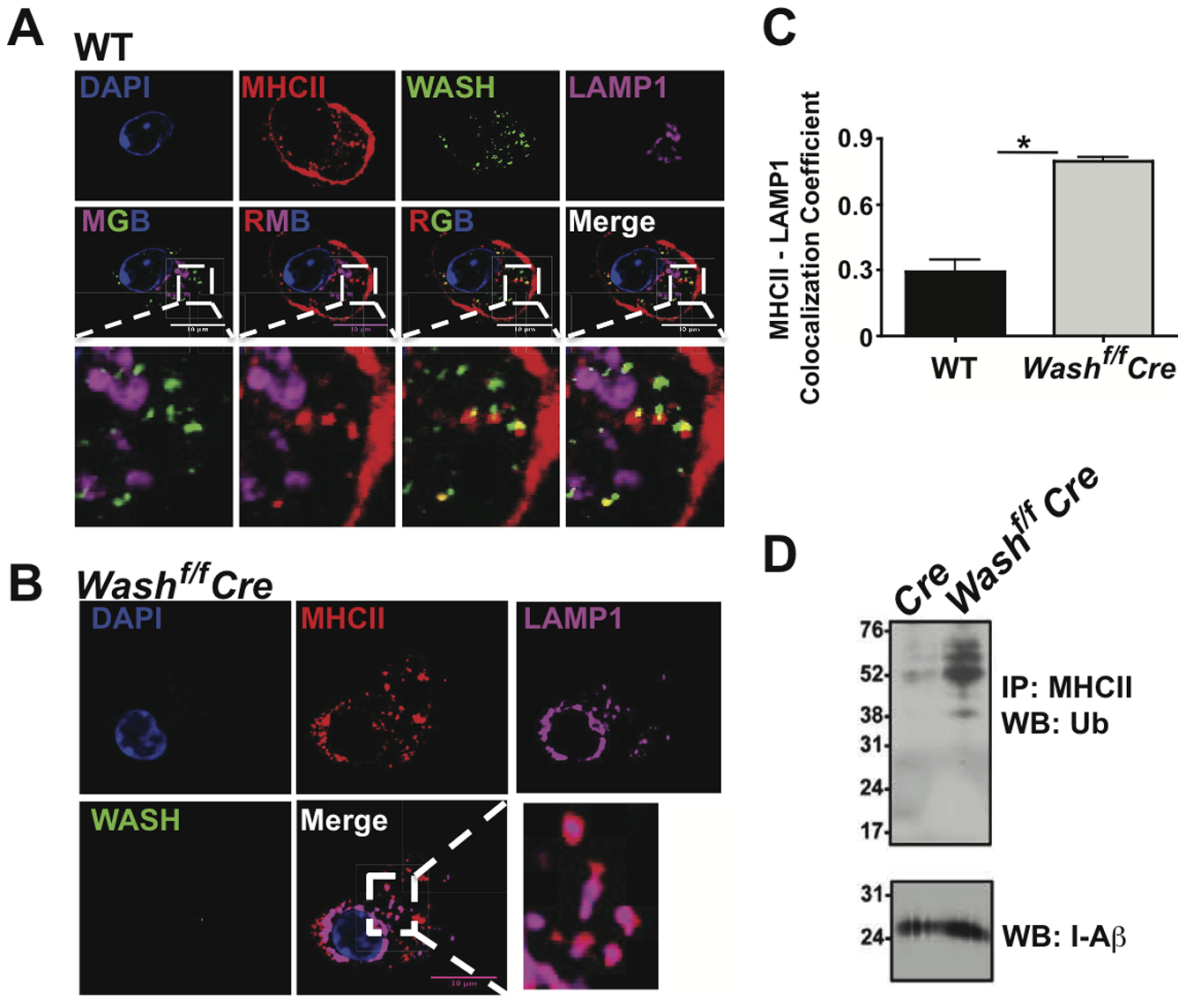
WASH prevents the localization of MHCII into lysosomes following endocytosis. BMDCs derived from (A) *Vav-Cre* and (B) *WASH^f/f^ Vav-Cre* were cultured with an antibody against MHCII following the endocytosis assay then fixed and labeled with antibodies against WASH and LAMP1 for microscopic analysis. (C) Images from A and B were analyzed for MHCII co-localization with LAMP1 using Pearson's co-localization coefficient in ZEN (Carl Zeiss). Zoomed images are demarcated by the white box and dashed lines in the adjacent images. For each condition, >20 individual cells were imaged. Images were collected with 100× oil objective. Scale bars, 10 µm. Bars represent mean ≥ SEM. Horizontal lines indicate statistical comparison between indicated groups, *p≤0.05. (D) Ubiquitinated MHCII was detected in BMDCs by immunoprecipitation of total MHCII followed by immunoblot for ubiquitin (Ub). Blots were subsequently stripped and reprobed with I-A^b^ antibody as a loading control.

The data presented above suggests that recycling of MHCII in endosomes involves WASH and retromer. Recently, we reported that the T cell receptor (TCR) and other molecules expressed by CD4^+^ T cells require WASH for efficient trafficking out of the recycling endosomal network [8], [11], [12], [14], [15]. In the absence of WASH, these receptors were found to accumulate in the lysosome and were degraded. If MHCII recycling is occurring, WASH and MHCII should be co-localizing together separate from the lysosome. To confirm if WASH indeed plays a role in the prevention of MHCII degradation, we analyzed MHCII localization with lysosomes in wild type and WASH-deficient DCs. Using antibodies against LAMP1, a lysosome marker, we observed that WASH and MHCII are co-localized, but LAMP1 is noticeably absent from the colocalized puncta (Figure 2A and zoom). In contrast, MHCII can be seen co-localizing with LAMP1 in WASH-deficient DCs (Figure 2B and zoom). Analysis of the MHCII and LAMP1 puncta confirms that in the absence of WASH, there are fewer MHCII-positive puncta and MHCII co-localization with LAMP1 in large intracellular puncta increases significantly (Figure 2D). Taken together these data suggest that WASH prevents MHCII from accumulating in lysosomes, thus allowing the recycling of the receptor to the surface. To confirm that the localization of surface-derived MHCII with WASH is specific to endosomal recycling, IF was performed using the endocytosis assay. We observed similar localization of MHCII with WASH (Figure 3A) and MHCII/WASH localization remains separate from LAMP1 stained lysosomes (Figure 3A). Again, in WASH-deficient DCs MHCII can be seen co-localizing with LAMP1 (Figure 3B and 3C).

### WASH-deficiency leads to hyperubiquitination of MHCII

The reduced MHCII cell surface retention and rapid degradation of MHCII observed in WASH-deficient DCs suggests that MHCII may accumulate in endosomes where it can be ubiquitinated by March1 and marked for degradation by entry into multivesicular bodies. To assay MHCII ubiquitination, we immunoprecipitated MHCII from control and WASH-deficient DCs prior to performing western blots for ubiquitin. In WASH-deficient DCs, the proportion of ubiquitinated MHCII relative to total levels was dramatically higher than in control DCs, despite the fact that similar levels of total MHCII were detected by immunoprecipitation in both genotypes (Figure 3D). Taken together, our observations in WASH-deficient DCs indicate that inefficient MHCII recycling from endosomal compartments promotes MHCII ubiquitination, leading to its rapid degradation in the lysosome.

### WASH-deficiency leads to rapid degradation of MHCII

**Figure 4:**
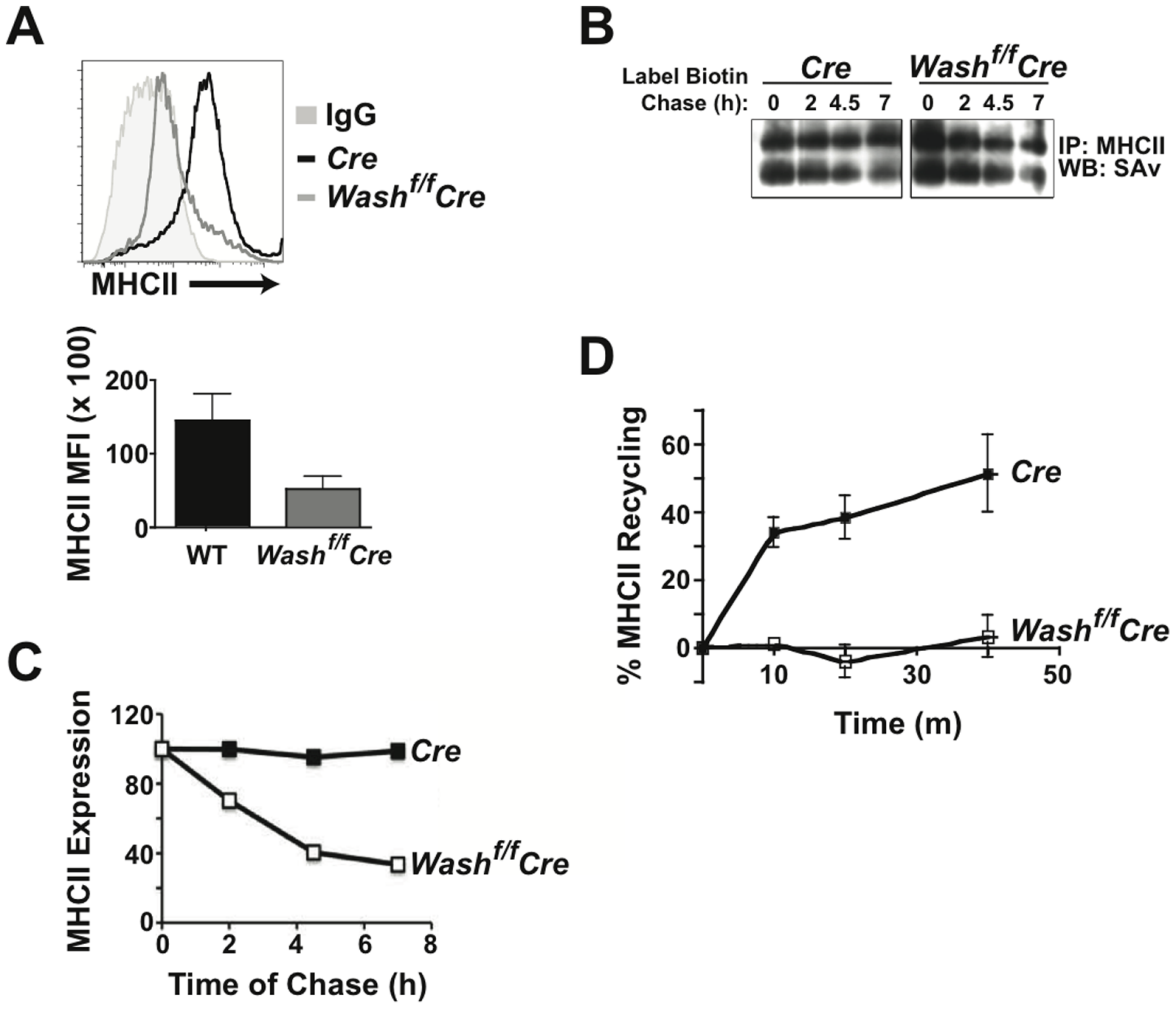
WASH-deficiency in DCs leads to polyubiquitination of MHCII and its accelerated degradation. (A) BMDCs from *WASH^f/f^* Vav-Cre and control *Vav-Cre* mice were antibody stained for surface MHCII and analyzed using flow cytometry. Histograms and the bar graph are representative of six separate experiments. (B) *WASH^f/f^ LysM-Cre* mice and control *LysM-Cre* mice BMDCs were surface labeled with biotin and chased in culture for the indicated time points. After chase, cells were lysed to immunoprecipitate MHCII. Immunoprecipitates were then resolved by PAGE and processed by immunoblot to detect remaining biotinylated MHCII with streptavidin (SAv). (C) Decay of surface labeled MHCII over time was measured by densitometry of immunoblots. (D) Percent MHCII recycling was measured on *WASH^f/f^ Vav-Cre* and control *Vav-Cre* BMDCs using acid stripping as described in the Materials and Methods. Data are representative of three separate experiments. Bars represent mean ≥ SEM, *p≤0.05.

We next examined the overall expression of MHCII on the surface of WASH-deficient DCs compared to control DCs. Using antibodies against I-A^b^ (murine MHCII expressed on DCs from C57BL/6 mice) and flow cytometry analysis, we found that WASH-deficient DCs exhibit surface expression of MHCII 2-logs below that of control DCs (Figure 4A). This suggests that WASH is responsible for maintaining MHCII surface expression. Next, to measure MHCII degradation kinetics, we surfaced-labeled control DCs and WASH-deficient DCs with biotin and chased for up to 7 hours. After the chase, cells were lysed and biotinylated MHCII was immunoprecipitated and quantification by streptavidin immunoblot was performed. We observed that MHCII is degraded in WASH-deficient DCs at a more rapid rate than in controls (Figure 4B). After a 7-hour chase, WASH-deficient DCs retained only 40% of surface labeled MHCII, while controls retained 90% (Figure 4C). These results suggest that WASH is required for efficient MHCII recycling back to the plasma membrane and indicate that WASH-dependent trafficking likely protects MHCII from premature lysosomal degradation.

### MHCII recycling is absent in WASH-deficient DCs

To determine if WASH is directly responsible for MHCII recycling, we used a recycling assay adapted from Sullivan *et al*. [8], [16]. BMDCs from *Wash^fl/fl^* and control Cre^+/+^ mice were surface stained for I-A^b^, the antibody was internalized and subsequently stripped following incubation after the indicated times (Figure 4D). Using flow cytometry to measure the mean fluorescence intensity (MFI) we determined the surface recycling difference between control DCs and WASH-deficient DCs. If recycling remained intact, the internalized antibody would return to the surface and be removed by the stripping buffer, leading to a reduction in fluorescence. As expected, the control DCs exhibited a reduction in I-A^b^ fluorescence whereas the WASH-deficient DCs demonstrated a minimal reduction in I-A^b^ fluorescence (Figure 4D). These data suggest that WASH is involved in the regulation of MHCII recycling and that in the absence of WASH, MHCII undergoes limited recycling compared to control DCs.

### WASH is required for MHCII antigen presentation

**Figure 5:**
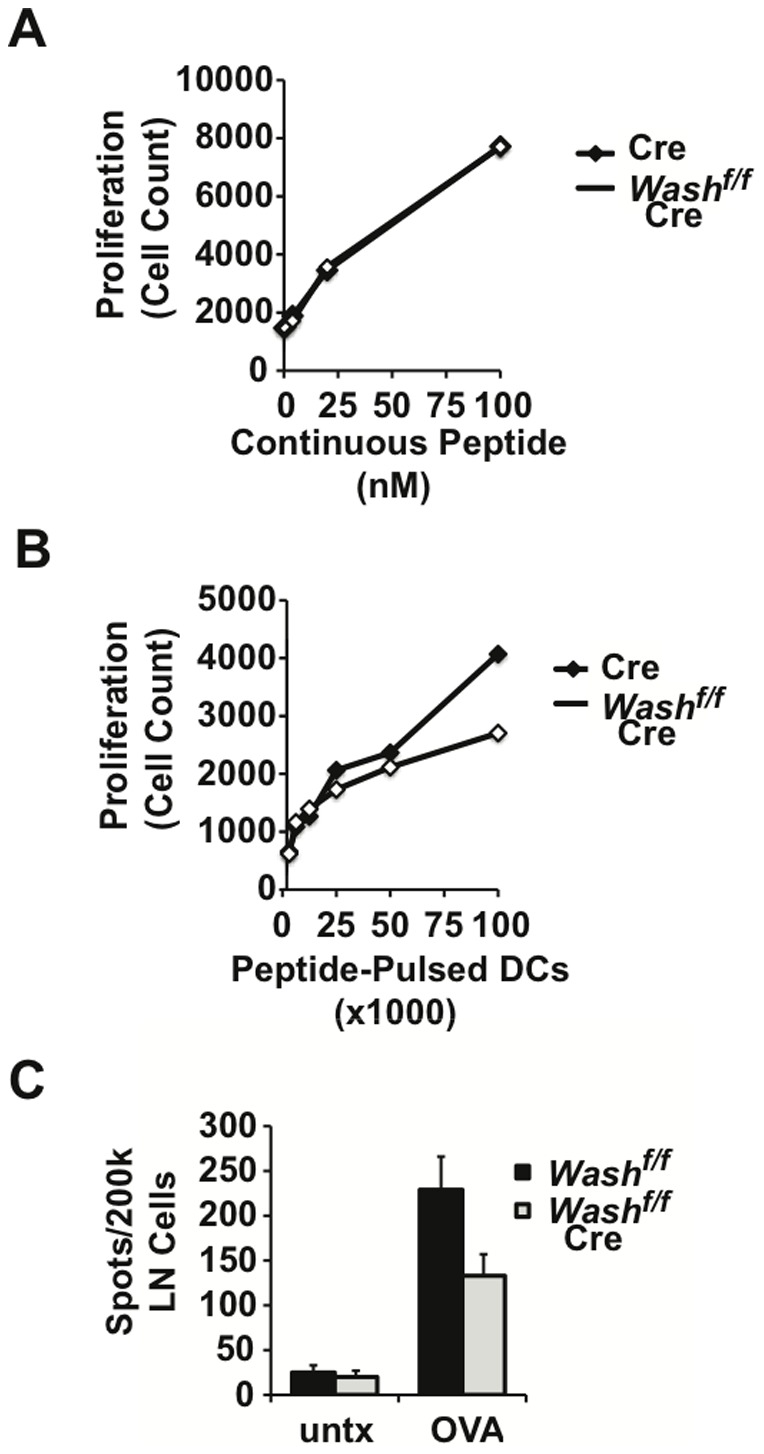
WASH is required for efficient antigen presentation and T cell priming. (A) BMDCs from *WASH^f/f^ LysM-Cre* mice and control *LysM-Cre* mice were cultured with OT-II T cells and ovalbumin-derived peptide antigen at the indicated doses. After three days in culture, T cell proliferation was determined by flow cytometry and cell count. (B) Alternatively, BMDCs were first pulsed with peptide antigen, then washed and cultured at the indicated cell numbers with OT-II T cells. T cell proliferation was determined by flow cytometry and cell count after three days. (C) *WASH^f/f^ CD11c-Cre* mice and control *WASH^f/f^* mice were immunized by subcutaneous injection of ovalbumin peptide in CFA. Seven days later, draining lymph nodes were harvested and restimulated *in vitro* with ovalbumin peptide. Antigen-specific T cells producing IL-2 upon restimulation were enumerated by ELISPOT.

The loss of recycling, hyperubiquitination, and rapid degradation of MHCII that we observed in WASH-deficient DCs predicts that WASH is required for efficient antigen presentation to T cells. To assay antigen presentation, we cultured control and WASH-deficient DCs with a constant dose of ovalbumin peptide and wild type OT-II T cells bearing a transgenic TCR specific for ovalbumin peptide loaded on I-A^b^. After three days in culture, OT-II T cell proliferation was measured to determine the efficiency of MHCII antigen presentation by DCs. When peptide was continuously present throughout the culture period, both control and WASH-deficient DCs were equally efficient at inducing wild type OT-II T cell proliferation (Figure 5A). This finding is consistent with the notion that, even if MHCII half-life is reduced in WASH-deficient DCs, newly synthesized MHCII can be loaded with peptide to replace pMHCII complexes that have been degraded. However, if antigen is limited, and exposure of DCs to antigen is restricted to a brief encounter, the stability/half-life of pMHCII complexes becomes a critical rate-limiting step for T cell activation. We therefore performed an experiment in which control and WASH-deficient DCs were pulsed with ovalbumin peptide and then thoroughly washed to remove excess antigen. Under these conditions, when antigen was limited, control DCs outperformed WASH-deficient DCs in stimulating OT-II T cell proliferation (Figure 5B).

To determine if WASH is required for MHCII antigen presentation and CD4 T cell priming *in vivo*, we employed a standard immunization protocol. Here, *Wash^fl/fl^* controls and *Wash^fl/fl^* CD11c-Cre mice were immunized in the footpad with ovalbumin peptide (OT-II). On day 7, we used IL-2 ELISPOT to enumerate antigen-specific CD4^+^ T cells in the draining lymph node. Consistent with our finding that WASH is required for MHCII antigen presentation *in vitro*, *Wash^fl/fl^* CD11c-Cre mice displayed a 40% reduction in frequency of IL-2 producing, antigen-specific T cells relative to control mice (Figure 5C). Thus, lineage specific deletion of WASH in DCs impairs CD4^+^ T cell priming *in vivo*. Based on these findings, we conclude that WASH-mediated trafficking of MHCII is critical for antigen presentation when antigen is limiting. In this context, endogenous DCs *in vivo* encounter antigen in a spatially and temporally restricted manner, thus the half-life of pMHCII complexes is a critical factor in priming CD4^+^ T cell responses.

### WASH-deficiency attenuates experimental autoimmune encephalitis

**Figure 6:**
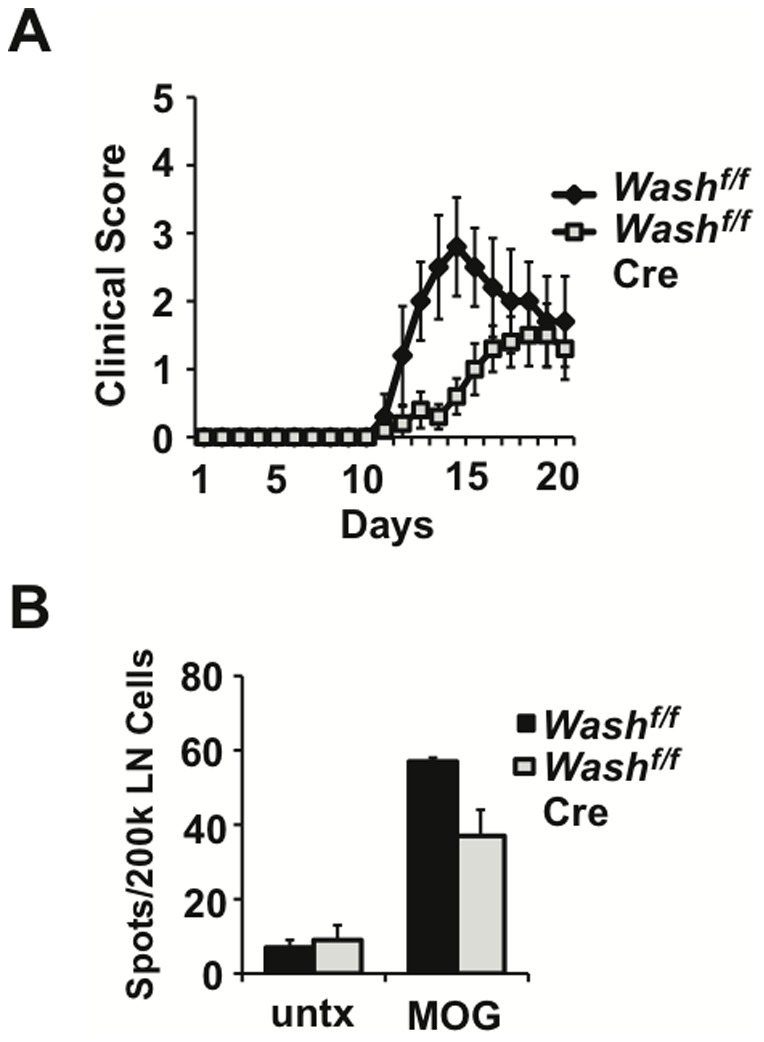
WASH-deficiency impairs priming of autoreactive T cells and attenuates disease progression in EAE. (A) *WASH^f/f^ CD11c-Cre* mice and control *WASH^f/f^* mice were immunized by subcutaneous injection with MOG peptide in CFA to induce experimental autoimmune encephalitis (EAE). Disease progression was monitored over time using the following scoring system: grade 1 = Tail weakness, grade 2 =  hind limb weakness sufficient to impair righting, grade 3 =  one limb plegic, grade 4 =  hind limb paralysis, grade 5 =  moribund. (B) Mice were immunized with MOG peptide as above and sacrificed at day seven. Draining lymph nodes were harvested and restimulated *in vitro* with MOG peptide to enumerate antigen-specific T cells by IL-2 ELISPOT.

Having established the requirement for WASH in CD4^+^ T cell priming *in vivo*, we sought to determine whether WASH is also required for the elicitation of encephalitogenic CD4^+^ T cells in experimental autoimmune encephalitis (EAE). Accordingly, *Wash^fl/fl^* controls and *Wash^fl/fl^* CD11c-Cre mice were immunized with myelin oligodendrocyte glycoprotein peptide 35–55 (MOG35-55) and monitored for development of clinical signs of disease. In this model of EAE, control mice develop an ascending paralysis starting at day 14 post-immunization, which progresses through day 21. However, in *Wash^fl/fl^* CD11c-Cre mice, disease onset was delayed and severity was attenuated (Figure 6A). At the peak of disease, control mice displayed infiltration of mononuclear cells into the lumbar spinal cord, a finding that was significantly reduced in *Wash^fl/fl^* CD11c-Cre mice (unpublished observation). To determine if attenuation of EAE observed in *Wash^fl/fl^* CD11c-Cre mice is associated with defective CD4^+^ T cell priming, we enumerated MOG-specific IL-2 producing T cells by ELISPOT at day 7-post immunization, the peak of the T cell response. We found that *Wash^fl/fl^* CD11c-Cre mice showed a significant reduction of autoreactive T cells compared to controls (Figure 6B). Thus, attenuation of EAE in *Wash^fl/fl^* CD11c-Cre mice can be attributed, at least in part, to defective CD4^+^ T cell priming. This finding is consistent with the notion that WASH is required for efficient recycling of MHCII to promote productive interactions with antigen-specific T cells during antigen presentation.

## Discussion

Turnover of the pMHCII complexes in DCs is a highly regulated process that strictly governs the efficiency of antigen presentation [8], [15], [17]–[23]. To effectively sample environmental antigens, immature DCs actively synthesize newly formed MHCII molecules that are continuously loaded with peptide antigen [8], [29]–[31]. Once loaded, pMHCII complexes briefly recycle from the plasma membrane through the endosomal system where March1 ubiquitinates MHCII, thus tagging the complex for lysosomal degradation [15], [20], [22]. The relatively short half-life of pMHCII complexes in immature DCs is essential to promote the continuous display of newly acquired antigenic peptides on the plasma membrane [8], [18], [19]. Thus, while a short pMHCII half-life promotes antigen sampling in immature DCs, the half-life of pMHCII must be finely tuned to optimally balance rapid antigen sampling with extended antigen presentation. In this context, we have previously shown that impaired pMHCII recycling results in a marked reduction in its half-life and a corresponding decrease in efficiency of antigen presentation to CD4^+^ T cells [17], [20]–[23]. By identifying the critical role of MHCII recycling in antigen presentation and T cell priming, our previous work highlights the importance of deciphering the molecular mechanisms directing MHCII trafficking. Here we identify WASH and the retromer component VPS35 as critical determinants of endosomal recycling in DCs that regulate pMHCII half-life and antigen presentation to CD4^+^ T cells.

While the function of retromer components appears to be highly conserved across phylogeny, we present evidence that DCs use the retromer to perform a unique and specialized function in MHCII recycling. In DCs, MHCII endocytosed from the cell surface and the retromer-associated WASH complex converge in the endosomal compartment. Accordingly, WASH acts as a critical mediator of receptor trafficking by coupling the retromer complex with the actin cytoskeleton. In this context, the sorting nexin retromer subunits mediate endosomal tubulation, while the vacuolar protein sorting subunits confer cargo specificity, and WASH is thought to direct actin polymerization required for normal endosome structure and domain organization leading to efficient receptor trafficking [7], [9], [15], [28], [32]–[35]. Importantly, here we identify a novel mechanism in which DCs employ the evolutionarily conserved WASH and retromer machinery for MHCII recycling thus allowing antigen sampling, loading and presentation resulting in optimal T cell activation.

The observation that DCs require WASH and VPS35 for cell surface retention of MHCII demonstrates their role in recycling. Furthermore, in the presence and absence of the Golgi transport inhibitor Brefeldin-A, MHCII surface levels are retained in control cells, suggesting that the retromer bypasses the Golgi and recycles MHCII directly back to the plasma membrane. The notion that DCs utilize the retromer to route MHCII directly to the plasma membrane from endosomes represents a novel adaptation of the retromer. Notably, a precedent exists for the role of retromer subunits in endosome to plasma membrane recycling [19], [24], [27], [28]. Recently, HEK293 cells were shown to utilize WASH and SNX27 for fast recycling of the β2-adrenergic receptor and resensitization of signaling [23], [24]. Moreover, SNX27 was found to not only interact with distinct cargo, but also make direct contacts with VPS26 and a component of the WASH complex to facilitate their trafficking via the retromer [27], [30]. Whether SNX27 or another sorting nexin is involved in binding MHCII and promoting its subcellular re-routing via WASH and retromer remains to be determined.

Trafficking of plasma membrane proteins between recycling and degradative compartments is essential for regulating their half-life and maintaining the spectrum of receptors displayed on the cell surface. We show that disrupting endosomal recycling by conditional deletion of WASH in DCs leads to a dramatic reduction in MHCII half-life, recycling and its accumulation in polyubiquitinated form. Presumably, impaired recycling traps MHCII in late endosomes where it encounters the E3 ubiquitin ligase MARCH1 [18]–[20], [24], [36], [37]. Following ubiquitination of the MHCII βchain, it is thought that the ESCRT complex transports MHCII into luminal vesicles that are subsequently delivered to lysosomes for degradation. Interestingly, it has been shown that unstable MHCII dimers accumulate in the lysosome[47]. It is suggested that in the acidic environment of the lysosome, these unstable MHCII molecules will unfold and aggregate, become associated with lysosomal chaperones, which will retain them in the lysosome ultimately leading to their degradation. Indeed, we show that the loss of WASH leads to an accumulation of MHCII in lysosomes and we have previously demonstrated that in the absence of WASH, both the endosomal and lysosomal systems collapse[9]. Thus, it remains possible that the lysosomal-accumulated MHCII molecules from the plasma membrane are unstable and thus being targeted for degradation instead of being retained in the MHC class-II-containing compartments for peptide loading.

As a consequence of impaired recycling and rapid degradation of MHCII, WASH- deficient DCs fail to efficiently present antigen to T helper cells. Limiting antigen exposure to DCs by a brief pulse *in vitro* revealed the requirement for WASH in inducing proliferation of antigen-specific T cells. Moreover, conditional deletion of WASH in the DC lineage impairs T cell priming *in vivo* and attenuates the clinical course of EAE. Similarly, we have previously shown that impairment of MHCII recycling by disruption of the ITAM signaling pathway in DCs prevents induction of EAE, at least in part due to defective T cell priming [23], [38], [39]. Taken together, we have identified a critical requirement for WASH and the retromer in maintaining a stable pool of pMHCII complexes on the surface of DCs for priming T cell responses. Thus, DCs utilize the evolutionarily conserved WASH and retromer complexes to complement their specialized antigen presenting capacity.

## Supporting Information

Figure S1 **Endocytosed MHCII transits through the early endosome.** (A) BMDCs derived from *Vav-Cre* mice were cultured with an antibody against MHCII following the endocytosis assay then fixed and labeled with antibodies against WASH and EEA1 for microscopic analysis. (B) Images from *Vav-Cre* and (B) *WASH^f/f^ Vav-Cre* were A were analyzed for MHCII co-localization with EEA1 in Cre and Vav using Pearson's co-localization coefficient in ZEN (Carl Zeiss). Zoomed images are demarcated by the white box and dashed lines in the adjacent images. For each condition, >20 individual cells were imaged. Images were collected with 100× oil objective. Scale bars, 10 µm. Bars represent mean ≥ SEM. *p≤0.05.
(TIFF)

Figure S2 **Expression of characteristic cell surface markers in BMDC cultures.** BMDCs from *Wash^f/f^* LysM-Cre^+/+^ mice and control LysM-Cre^+/+^ mice were stained with the indicated antibodies and analyzed by FACS. Although WASH appears to regulate expression of several surface markers, proliferation and survival of *Wash^f/f^* LysM-Cre^+/+^ BMDCs cultured in GM-CSF were similar to wild type and LysM-Cre^+/+^ controls.
(TIFF)
