# Supplementary figures and images for: Dendritic Cells Utilize the Evolutionarily Conserved WASH and Retromer Complexes to Promote MHCII Recycling and Helper T Cell Priming
Source: PLoS One. 2014 Jun 2;9(6):e98606. doi: 10.1371/journal.pone.0098606 (PMC4041763; doi:10.1371/journal.pone.0098606)

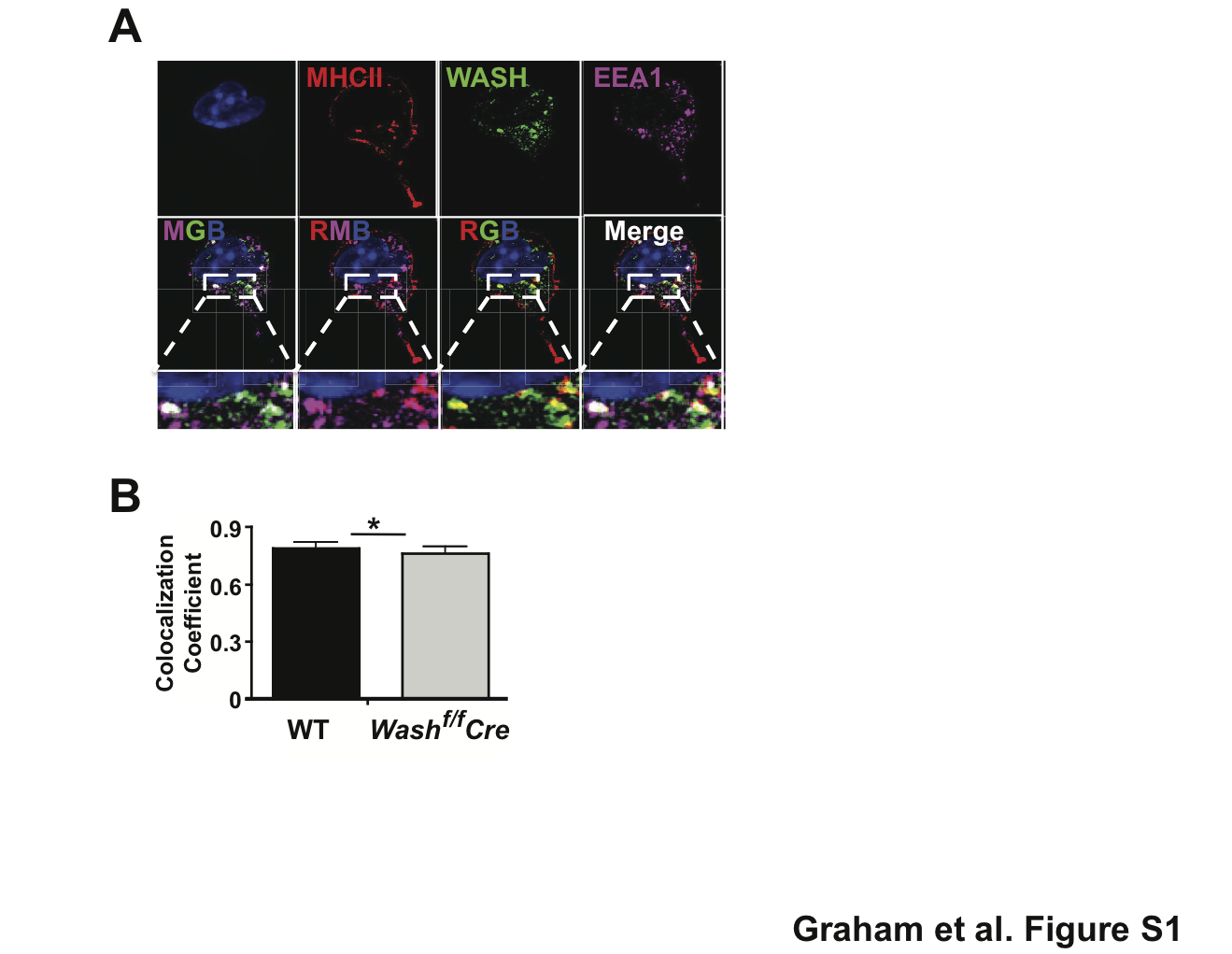

Supplement: Figure S1 — Endocytosed MHCII transits through the early endosome. (A) BMDCs derived from Vav-Cre mice were cultured with an antibody against MHCII following the endocytosis assay then fixed and labeled with antibodies against WASH and EEA1 for microscopic analysis. (B) Images from Vav-Cre and (B) WASHf/f Vav-Cre were A were analyzed for MHCII co-localization with EEA1 in Cre and Vav using Pearson's co-localization coefficient in ZEN (Carl Zeiss). Zoomed images are demarcated by the white box and dashed lines in the adjacent images. For each condition, >20 individual cells were imaged. Images were collected with 100× oil objective. Scale bars, 10 µm. Bars represent mean ≥ SEM. *p≤0.05. (TIFF) [file pone.0098606.s001.tif]

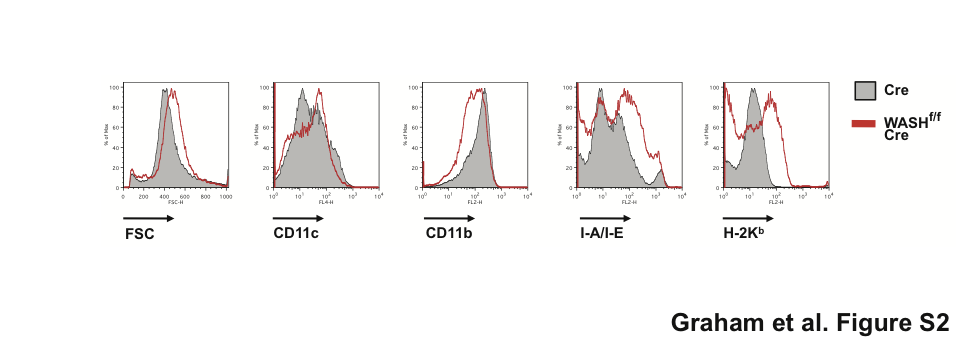

Supplement: Figure S2 — Expression of characteristic cell surface markers in BMDC cultures. BMDCs from Washf/f LysM-Cre+/+ mice and control LysM-Cre+/+ mice were stained with the indicated antibodies and analyzed by FACS. Although WASH appears to regulate expression of several surface markers, proliferation and survival of Washf/f LysM-Cre+/+ BMDCs cultured in GM-CSF were similar to wild type and LysM-Cre+/+ controls. (TIFF) [file pone.0098606.s002.tif]
